# An association between fibroblast growth factor 21 and cognitive impairment in iron-overload thalassemia

**DOI:** 10.1038/s41598-021-87597-x

**Published:** 2021-04-13

**Authors:** Wasan Theerajangkhaphichai, Jirapas Sripetchwandee, Sirawit Sriwichaiin, Saovaros Svasti, Nipon Chattipakorn, Adisak Tantiworawit, Siriporn C. Chattipakorn

**Affiliations:** 1 Division of Hematology, Department of Internal Medicine, Faculty of Medicine, Chiang Mai University, Chiang Mai, 50200 Thailand; 2 Neurophysiology Unit, Cardiac Electrophysiology Research and Training (CERT) Center, Faculty of Medicine, Chiang Mai University, Chiang Mai, 50200 Thailand; 3 Department of Physiology, Faculty of Medicine, Chiang Mai University, Chiang Mai, 50200 Thailand; 4 Thalassemia Research Center, Institute of Molecular Biosciences, Mahidol University, Nakhon Pathom, 73170 Thailand; 5 Department of Oral Biology and Diagnostic Sciences, Faculty of Dentistry, Chiang Mai University, Chiang Mai, 50200 Thailand

**Keywords:** Medical research, Translational research, Cognitive ageing, Neuroendocrine diseases

## Abstract

Although an increased fibroblast growth factor 21 (FGF21) level was related to mild cognitive impairment (MCI) in metabolic syndrome patients, any association regarding FGF21 and MCI in thalassemia patients as well as mechanistic insight are questionable. Therefore, the objectives of this study were: (1) to investigate the prevalence and associative risk factors of MCI in thalassemia patients, (2) to evaluate the association between levels of FGF21 and MCI in thalassemia patients, and (3) to investigate brain FGF21 signaling in iron-overload thalassemia. Thalassemia patients were enrolled onto the study (n = 131). Montreal cognitive assessment (MoCA) was used to determine cognitive performance. Plasma FGF21 level was determined in all patients. Iron-overload β-thalassemic (HT) mice were used to investigate brain FGF21 level and signaling, the expression of synaptic proteins, and Alzheimer’s like pathology. We found that 70% of thalassemia patients developed MCI. FGF21 level was positively correlated with the MCI. Interestingly, brain FGF21 resistance, as indicated by increased brain FGF21 levels with impaired FGF21 signaling, was found in iron-overload HT mice. The reduced synaptic protein expression and increased Alzheimer’s like pathology were also observed. These suggest that FGF21 may play a role in MCI in thalassemia patients.

## Introduction

Although most of thalassemia patients require adequate blood transfusion to lengthen the life span^1,2^, iron overload is the most common complication in thalassemia patients resulting from multiple blood transfusions and excessive increased intestinal iron absorption^3,4^. Not only an abnormality in major organs, particularly in the heart and liver, neurocognitive dysfunction has also been reported^5–7^. Our recent study in iron-overloaded thalassemic mice indicated neurotoxicity as shown by brain iron overload, brain oxidative stress and inflammation, hyperactivation of glia, brain mitochondrial abnormalities, and Alzheimer's like pathology^8^. However, there was a clinical study showing that an intense iron accumulation is specifically detectable in choroid plexus in five thalassemic patients without any intervention such as iron chelation^9^ and this has been confirmed by the most recent brain MRI study^10^. Taken together, these inconsistent findings suggest the importance of underlying mechanisms responsible for brain iron overload and neurotoxicity from iron overload in thalassemic patients.

Mild cognitive impairment (MCI) is an early phase of neurological disorder which could further progress to dementia, in particular into Alzheimer’s disease^11^. Early detection of cognitive impairment by determining MCI condition in the neurodegeneration would provide the benefit of potential prevention of further impairment of cognitive function. Although several studies reported the observation of MCI in the pathological conditions including cerebral infarction and diabetes^12,13^, the relationship between iron overload status and the risk of MCI in thalassemia patients has never been reported.

Fibroblast growth factor 21 (FGF21), one of the endocrine hormones that is mainly produced by the liver, has several functions including cell development and proliferation, wound healing, and the regulation of energy homeostasis^14,15^. Previous clinical reports of studies in metabolic syndrome (MetS) patients indicated that a high level of FGF21 was linked with a lower score of the MoCA test in MetS patients aged 65 and below^16^. Additionally, a recent study found that obese-insulin resistant rats have developed an impairment of brain FGF21 signaling with increased serum/brain FGF21 levels, and cognitive dysfunction, suggesting the occurrence of brain FGF21 resistance which might lead to cognitive decline in this condition^17^. Collectively, these findings imply that FGF21 might play a pivotal role in the pathophysiology of MCI. Nonetheless, any association of FGF21 in relation to the MCI of thalassemia patients as well as the underlying mechanism associated with FGF21 in cases of iron-overload have never been investigated.

Thus, the aims of the present study are: (1) to investigate the prevalence and associated risk factors of MCI in thalassemia patients, (2) to evaluate the clinical significance of FGF21 and associative risk factors of MCI in thalassemia patients, and (3) to investigate the mechanisms associated with FGF21 and iron-induced brain toxicity in thalassemic mice.

## Results

### Demographic data from thalassemia patients demonstrated a significant alteration in hematologic parameters and plasma FGF21 level in cases of MCI

Between November 2018 and November 2019, one-hundred and thirty-one thalassemia patients were enrolled onto the study. Sixty-seven percent of patients were female, and the mean age of participant was 35 ± 11 years. Most of patients were graduates with a bachelor’s degree (44%) and three patients had a master’s degree. The most common type of thalassemia was beta thalassemia/hemoglobin E disease which accounted for about two-thirds of patients, whereas homozygous beta-thalassemia and hemoglobin H disease patients totaled 20.6% and 16.8%, respectively. Splenectomies had been carried out in 48.9% of patients. The majority of the patients (60.3%) were TDT. The iron overload condition was present in 76.3% of patients and these had been treated with iron chelators. Deferiprone was the most common drug that was used in iron overload patients in this study. Mean hemoglobin was 7.2 ± 1.3 g/dl and mean hematocrit was 23.2 ± 4.9%. The median serum ferritin at the time of enrollment and maximum level of serum ferritin in the past 5 years were 1324 ng/ml and 1959 ng/ml, respectively (Table 1).

**Table 1:** Demographic data of thalassemia patients.

Patient characteristics	n = 131
**Gender, n (%)**	
Male	43 (32.8)
Female	88 (67.2)
Age (year)	35 ± 11
**Education, n (%)**	
Primary education	23 (17.5)
Junior high school	11 (8.4)
Senior high school	36 (27.5)
Bachelor’s degree	58 (44.3)
Master’s degree	3 (2.3)
**Thalassemia typing, n(%)**	
Hemoglobin H	22 (16.8)
Beta thalassemia/hemoglobin E	82 (62.6)
Homozygous beta-thalassemia	27 (20.6)
**Splenectomy, n(%)**	
Yes	64 (48.9)
No	67 (51.1)
**Transfusion dependent, n(%)**	
Yes	79 (60.3)
No	52 (39.7)
Hemoglobin (g/dl)	7.2 ± 1.3
Hematocrit (%)	23.2 ± 4.9
Serum ferritin (ng/ml)	1324 (1497)
Maximum level of serum ferritin in the past 5 years (ng/ml)	1959 (2221)
**Iron chelating agents, n(%)**	
Deferoxamine	8 (6.1)
Deferiprone	48 (36.6)
Deferasirox	11 (8.4)
Deferoxamine + deferiprone	21 (16.0)
Deferoxamine + deferasirox	12 (9.2)
No	31 (23.7)

Data from age, hemoglobin, hematocrit, and MoCA score were represented by mean ± SD, while data from serum ferritin and Maximum level of serum ferritin in the past 5 years were represented as median and IQR.

*MoCA* montreal cognitive assessment.

The mean MoCA score measuring cognitive performance was 23 ± 4 points from a total of 131 thalassemia patients (Table 1). Interestingly, the prevalence of MCI as indicated by MoCA score < 26 points was 70.2% (92 of 131 patients). Of these patients who exhibited MCI, median serum ferritin level and the maximum level of serum ferritin in the past 5 years were 1398 ng/ml and 2382 mg/ml, respectively (Table 2). A significant difference in maximum level of serum ferritin in the past 5 years between MCI- and normal cognitive function-patients was observed (Table 2). Not only was the serum ferritin significantly higher in comparison with patients within normal limits of cognitive function, the median FGF21 level in the plasma of MCI group was 385 pg/ml (Table 2). Regarding NTDT patients, patients with MCI had significantly higher maximum serum ferritin level within 5 years (mean difference = 879.046, *p* = 0.015), ALT (mean difference = 13.293, *p* = 0.005), and plasma FGF21 level (mean difference = 131.211, *p* = 0.022), when compared with patients with normal cognitive function. In addition, the serum ferritin and maximum serum ferritin within 5 years were significantly lower in NTDT than TDT patients. However, the number of patients with MCI (65.4% in NTDT vs 73.4% in TDT, *p* = 0.336) and the MoCA score (mean difference = 0.354, *p* = 0.633) between NTDT and TDT patients were not significantly different. These findings suggest that high levels of serum ferritin and plasma FGF21 may be associated with MCI in these thalassemia patients.

**Table 2:** Demographic data among normal and mild cognitive impairment group.

Variables	Normal (MoCA ≥ 26) n = 39	MCI (MoCA < 26) n = 92	p-value
Age (year)	31 (9)	37 (12)	**0.005**
**Thalassemia typing, n (%)**			0.698
Hemoglobin H	7	14	
Beta thalassemia/hemoglobin E	26	56	
Homozygous beta-thalassemia	6	21	
Hemoglobin H/CS	0	1	
Transfusion dependent, n (%)	21 (53.8)	58 (63.0)	0.325
Splenectomy, n (%)	20 (51.3)	44 (47.8)	0.717
Hemoglobin (g/dl)	7.31 ± 1.29	7.16 ± 1.40	0.561
Hematocrit (%)	24 ± 4	23 ± 5	0.701
Serum ferritin (ng/ml)	1122 (1967)	1398 (1431)	0.160
Maximum level of serum ferritin in the past 5 years (ng/ml)	1561 (2230)	2382 (2158)	**0.020**
FGF21 level (pg/ml)	250 (312)	385 (413)	**0.004**
BUN (mg/dl)	14 ± 12	15 ± 11	0.715
Creatinine (mg/dl)	0.66 ± 0.80	0.65 ± 0.41	0.925
eGFR (ml/min)	131 ± 28	121 ± 30	0.100
Total protein (g/dl)	8.17 ± 0.75	8.13 ± 0.62	0.763
Albumin (g/dl)	4.44 ± 0.49	4.34 ± 0.36	0.240
Globulin (g/dl)	3.73 ± 0.89	3.79 ± 0.77	0.711
AST (U/L)	34 ± 23	42 ± 26	0.101
ALT (U/L)	26 ± 22	35 ± 29	0.074

Data were represented by mean ± SD, whereas data from serum ferritin, maximum level of serum ferritin in the past 5 years, and FGF21 level were represented as median and IQR. Montreal Cognitive assessment.

*MCI* mild cognitive impairment, *FGF21* fibroblast growth factor 21, *BUN* blood urea nitrogen, *eGFR* estimated glomerular filtration rate, *AST* aspartate aminotransferase, *ALT* alanine aminotransferase.

Univariate analysis was used to analyze the possible factors related to the occurrence of MCI in thalassemia patients (Table 3). The results demonstrated that several factors including age (Odds ratio (OR) = 1.049, 95% Confidence interval (CI) 1.010–1.090), ln plasma FGF21 (OR = 2.107, 95% CI 1.317–3.371), and ln maximum serum ferritin in 5 years (OR = 1.806, 95% CI 1.084–3.007) were significantly associated with MCI. When data was analyzed using multivariate logistic regression analysis, age (OR = 1.507, 95% CI 1.004–1.112), plasma FGF21 (OR = 1.858, 95% CI 1.074–3.213), and patients educated to bachelor’s degree level and above (OR = 0.282, 95% CI 0.112–0.707) showed an independent association with MCI (Table 3). In contrast, other factors including sex, thalassemia type, splenectomy status, and transfusion-dependent status, were not associative risk factors of MCI of these patients (Table 3).

**Table 3:** Binary logistic regression analysis of association to determine the factors associated with mild cognitive impairment in thalassemia patients.

Variables	Univariate		Multivariate	
	Odds ratio (95% CI)	p-value	Odds ratio (95% CI)	p-value
Age (years)	1.049 (1.010–1.090)	**0.011**	1.057 (1.004–1.112)	**0.034**
Hemoglobin (g/dL)	0.922 (0.702–1.211)	0.558	1.150 (0.814–1.624)	0.428
ln FGF21	2.107 (1.317–3.371)	**0.002**	1.858 (1.074–3.213)	**0.027**
ln maximum serum ferritin in 5 years	1.806 (1.084–3.007)	**0.023**	1.441 (0.710–2.926)	0.312
**Sex**				
Male	1	Reference		
Female	1.127 (0.553–2.676)	0.626	1.759 (0.687–4.501)	0.239
**Education level**				
Below bachelor’s degree	1	Reference	1	Reference
Bachelor’s degree and above	0.261 (0.117–0.580)	**0.001**	0.282 (0.112–0.707)	**0.007**
**Thalassemia type**				
Alpha-thalassemia	1	Reference	1	Reference
Beta-thalassemia	1.123 (0.418–3.014)	0.818	2.236 (0.419–11.932)	0.346
**Splenectomy status**				
Non-splenectomy	1	Reference	1	Reference
Splenectomy	0.817 (0.412–1.842)	0.718	0.619 (0.222–1.727)	0.360
**Transfusion dependent status**				
NTDT	1	Reference	1	Reference
TDT	1.462 (0.685–3.122)	0.326	1.187 (0.385–3.661)	0.765

Age, hemoglobin, ln FGF21, ln maximum serum ferritin in 5 years, sex, education level, thalassemia type, splenectomy status, and transfusion dependent status were used in binary logistic regression analysis.

*FGF21* fibroblast growth factor 21, *NTDT* non-transfusion dependent thalassemia, *TDT* transfusion dependent thalassemia.

After dividing patients in this study into two groups based on mean hemoglobin (Hb) levels (7.2 mg/dL). We found that no significant difference of MoCA scores between two groups was observed (mean difference = 1.049, *p* = 0.147). Furthermore, factors that might be correlated with the cognitive function in each group were analyzed. The results showed that age (r = − 0.371, *p* = 0.003), and FGF21 (r = − 0.293, *p* = 0.021) were correlated with MoCA scores in the patients with Hb level higher than 7.2 mg/dL. For the patients with Hb lower than 7.2 mg/dL, only age was correlated with MoCA score (r = − 0.303, *p* = 0.011).

### *An *in vivo* study demonstrated mechanistic insight regarding the association of FGF21 and cognitive impairment*

**Figure 1:**
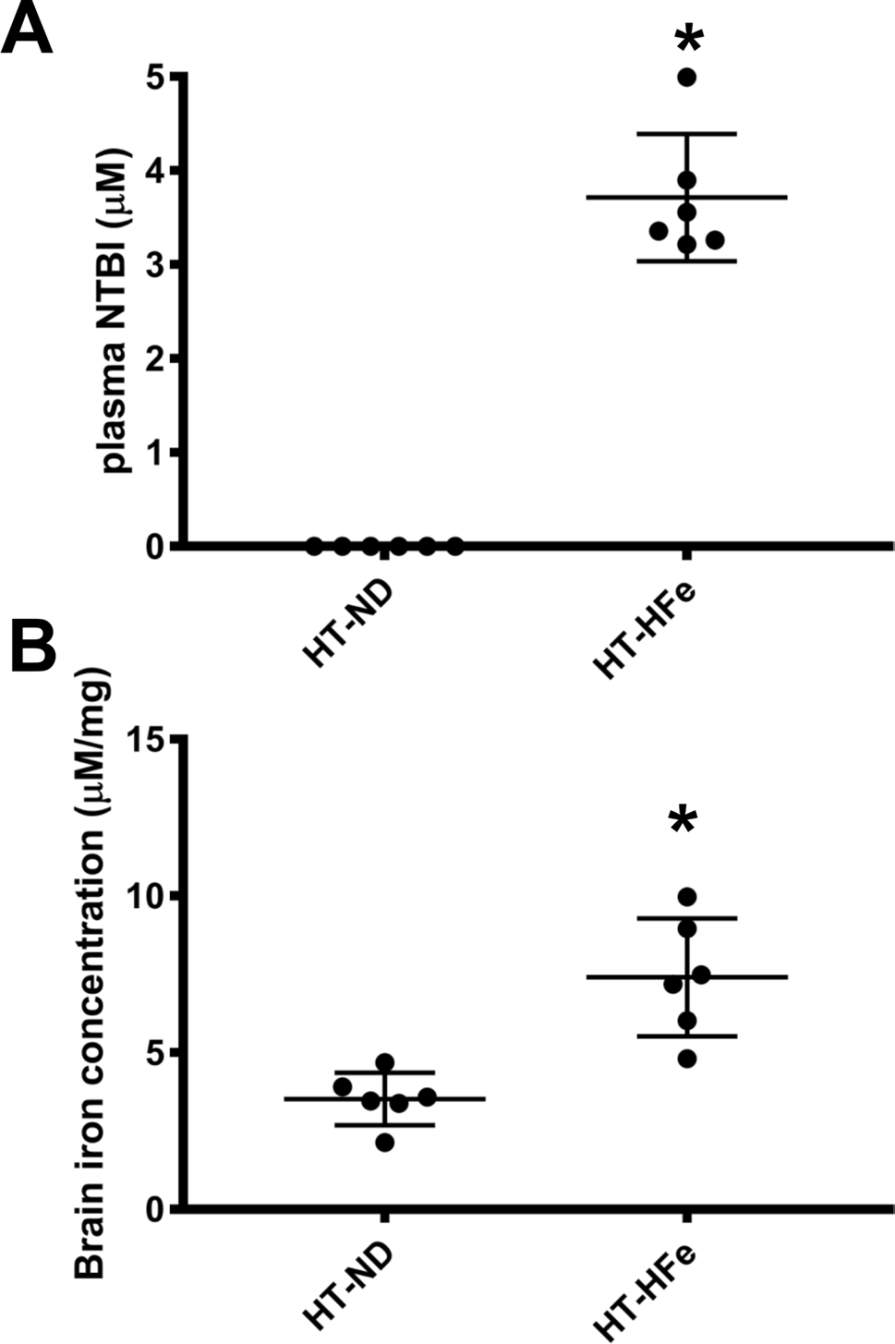
Plasma iron level (**A**) and brain iron concentration (**B**) of β-thalassemic mice fed with either normal diet or high-iron diet. *HFe* high-iron diet fed mice, *HT* heterozygous-β knockout, *ND* normal diet fed mice. *p < 0.05 vs. HT-mice fed with normal diet.

**Figure 2:**
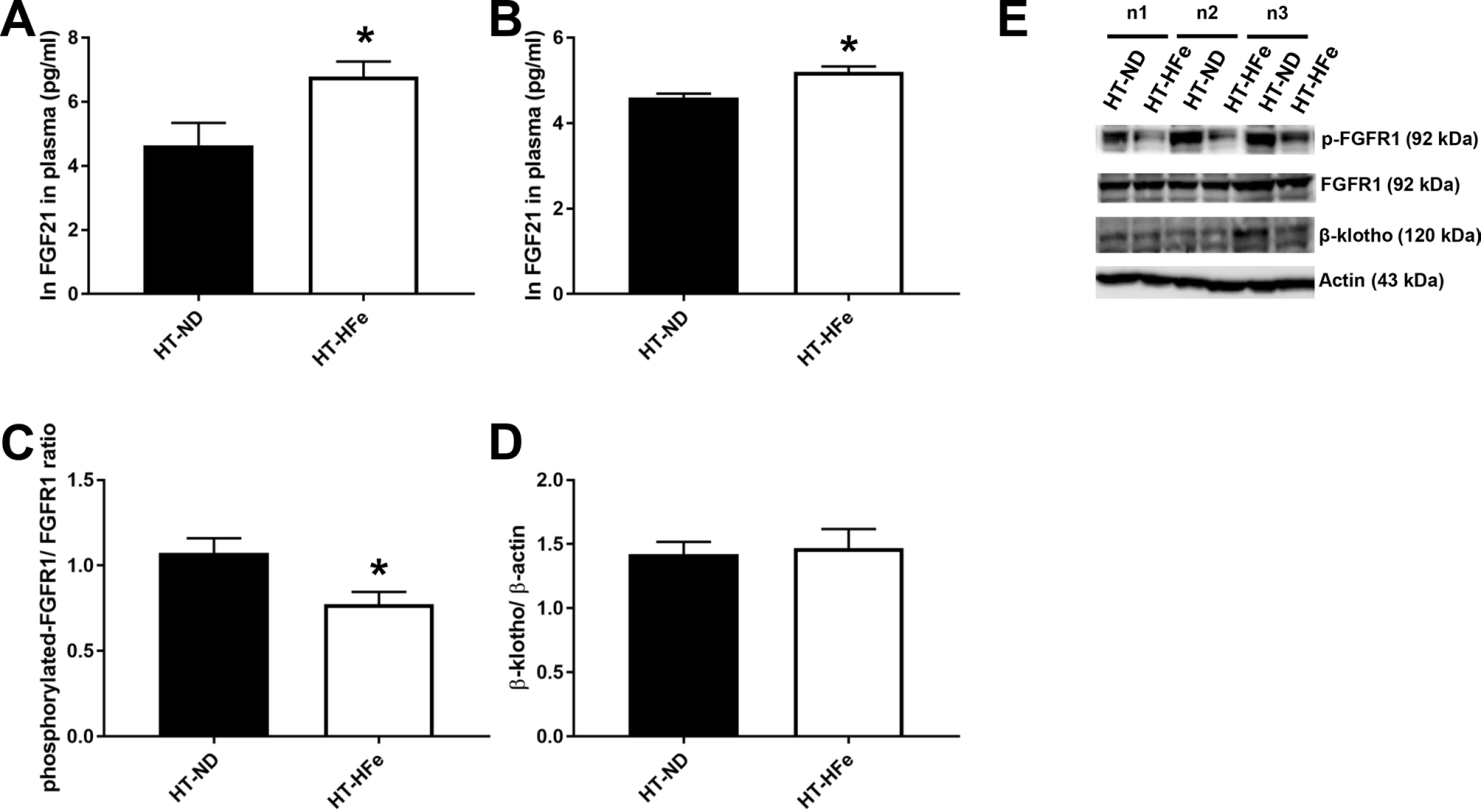
Plasma FGF21 level in plasma (**A**), brain FGF21 level (**B**), and brain FGF21 signaling cascades including a ratio of phosphorylated-FGFR1/FGFR1 (**C**) and β-klotho/actin (**D**) of β-thalassemic mice fed with either normal diet or high-iron diet. Three replicates of cropped representative bands for brain FGF21 signaling proteins are shown (**E**). In addition, full-length blots are presented in Supplementary Fig. 1. The samples derive from the same experiment and that gels/blots were processed in parallel. Actin was used as a loading control and the density of detected protein was divided by the density of actin from the sample. *FGF21* fibroblast growth factor 21, *FGFR1* fibroblast growth factor receptor 1, *HFe* high-iron diet fed mice, *HT* heterozygous-β knockout, *ND* normal diet fed mice. *p < 0.05 vs. HT-mice fed with normal diet.

To investigate the association among FGF21 level, brain FGF21 signaling pathway and the MCI condition, thalassemia mice fed on a HFe to promote systemic iron overload were used. The results indicated that HFe-fed mice expressed an upregulated plasma NTBI (Fig. 1A) with an elevated brain iron concentration in these mice, compared to the control group of ND-fed mice (Fig. 1B). Surprisingly, the levels of ln FGF21 in both the plasma and brain of HFe-fed mice were increased (Fig. 2A,B). Regarding the expression of FGF21 signaling proteins in brain, decreased brain FGF21 signaling was found in HFe-fed mice as shown by the reduction of phosphorylated-FGFR1/FGR1 expression (Fig. 2C), although there was no change in the expression of β-klotho (Fig. 2D). All cropped representative bands for brain FGF21 signaling proteins were shown in Fig. 2E. Additionally, full-length blots of FGF21 signaling proteins in brain were presented in Supplementary Fig. 1. Collectively, the upregulation of FGF21 level and the reduction in brain FGF21 signaling might imply that brain FGF21 resistance had developed in iron-overloaded thalassemic mice.

**Figure 3:**
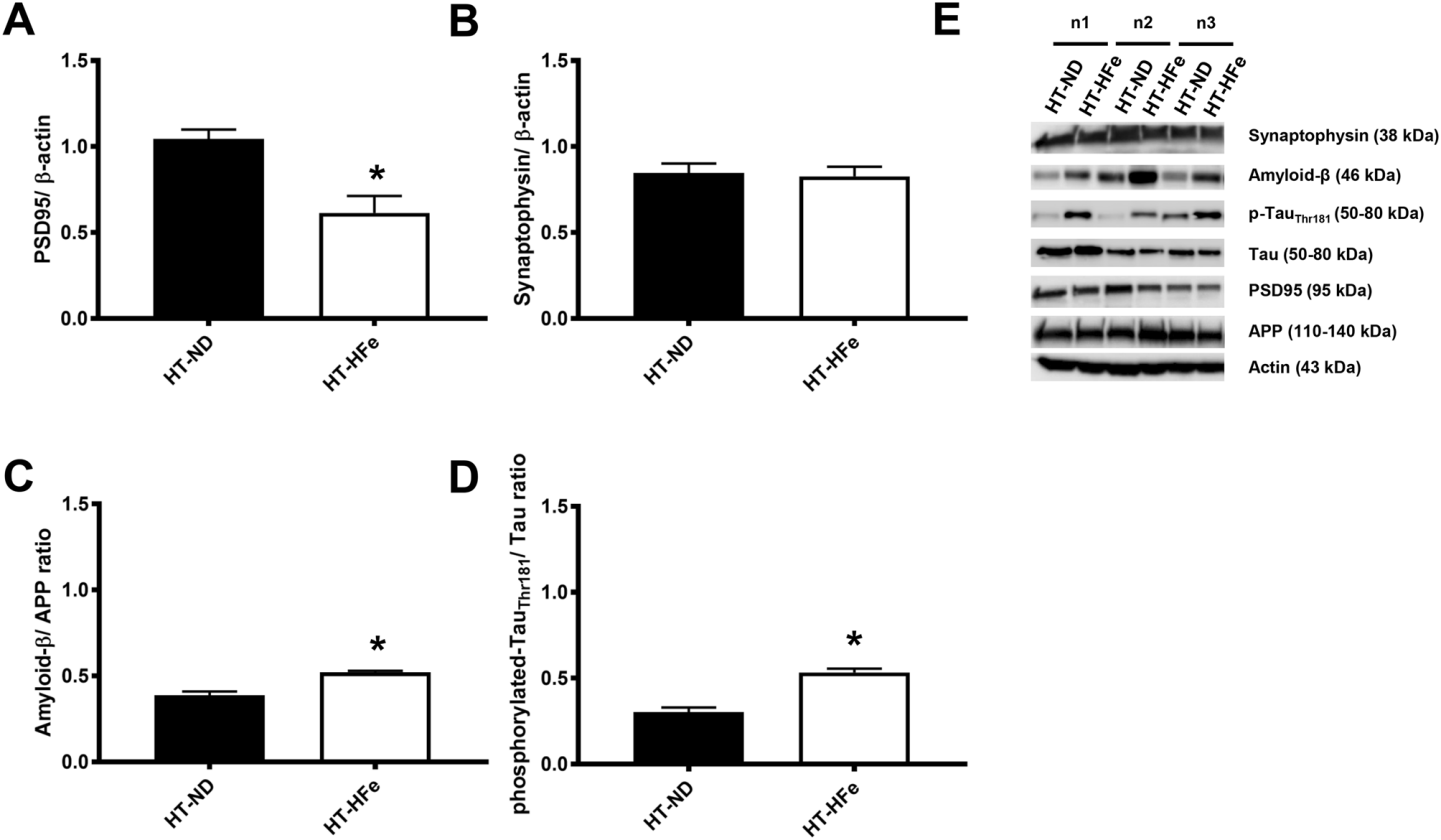
Brain synaptic proteins including PSD95 (**A**) and synaptophysin (**B**) and Alzheimer’s related proteins including the ratios of Amyloid-β/APP (**C**) and phosphorylated-Tau_Thr181_/Tau (**D**) of β-thalassemic mice fed with either normal diet or high-iron diet. Three replicates of cropped representative bands for brain FGF21 signaling proteins are shown (**E**). In addition, full-length blots are presented in Supplementary Fig. 2. The samples derive from the same experiment and that gels/blots were processed in parallel. Actin was used as a loading control and the density of detected protein was divided by the density of actin from the sample. *APP* amyloid precursor protein, *HFe* high-iron diet fed mice, *HT* heterozygous-β knockout, *ND* normal diet fed mice, *PSD95* post-synaptic density 95. *p < 0.05 vs. HT-mice fed with normal diet.

Not only was there alteration in FGF21 in the brain, but the results also showed that the expression of post-synaptic protein (PSD95) was decreased in HFe-fed mice, compared to that in ND-fed mice (Fig. 3A), although the level of pre-synaptic protein (synaptophysin) did not change (Fig. 3B). Results pertaining to Alzheimer’s like pathology showed that both Amyloid-β/APP (Fig. 3C) and the phosphorylated-Tau_Thr181_/Tau ratio in HFe-fed mice were up-regulated (Fig. 3D). All cropped representative bands for brain synaptic proteins and Alzheimer’s-like pathology were demonstrated in Fig. 3E. Furthermore, full-length blots of brain synaptic proteins and Alzheimer’s-like pathology were presented in Supplementary Fig. 2. These findings suggest that amyloid accumulation and tau-hyperphosphorylation were occurred in the brain of those mice.

These results from both thalassemia patients and thalassemia mice suggest that cognitive impairment in thalassemia patients possibly occurs in association with brain FGF21 resistance which was shown in thalassemia mice.

## Discussion

Major findings from this study are as follows: (1) MCI condition occurred in thalassemia patients, (2) hematologic parameters including serum ferritin level and maximum level of serum ferritin in the past 5 years were significantly increased in thalassemia patients with MCI, (3) clinically, plasma FGF21 level showed an independent association with MCI, (4) mechanistically, brain FGF21 resistance may be related to the alteration of brain synaptic protein and Alzheimer’s like pathology, and (5) MCI in thalassemia patients may be associated with FGF21 resistance.

Iron overload was the most common complication in cases of both TDT and NTDT. Currently, many iron-chelating agents are widely used in thalassemia patients with iron overload, leading to lengthening of the life span of thalassemia patients^2^. Although children with beta-thalassemia disease have cognitive deficits compared to their age-matched controls has been reported^18^, data on the prevalence and associative risk factors of cognitive impairment in thalassemia adults is limited. This study showed a high prevalence of 70.2% of MCI in Thai thalassemia patients therefore it is useful to investigate a possible correlation with risk factors associated with MCI in these patients.

Not only age and low-educational level of patients were associated with MCI in these patients, but also several hematologic parameters, particularly the maximum level of serum ferritin in the past 5 years. We used the mean maximum value of serum ferritin in the past 5 years since this value represents the highest value of ferritin which might be toxic to the vital organs, particularly in endocrine and brain, and may not be interfered by treatment with iron chelators. In addition, although total 7 out of 131 patients have been diagnosed as hepatitis C viral infection, a pathological condition that might affect the level of ferritin, patients who have been diagnosed with several factors which interfere the serum ferritin level including active infection, inflammation were excluded.

There were reports regarding a higher brain iron concentration with accelerated cognitive decline in iron-loaded mice^19^ and the brain iron accumulation in Alzheimer’s patients^20^. Surprisingly, we demonstrated that the maximum serum ferritin levels in the past 5 years, not the current serum ferritin levels, in thalassemia patients was significantly different between patients with MCI and normal cognition. Possible explanation may be that the majority of thalassemia patients with iron overload in this study were treated with iron-chelating agents, resulting in a reduction of current serum ferritin levels. Therefore, in this study the maximum serum ferritin in the past 5 years would represent iron overload status more effectively than current serum ferritin levels. These maximum serum ferritin levels over the past 5 years were also negatively associated with MoCA score, suggesting that an increase in this parameter may be used as one of the biomarkers for MCI in thalassemia patients.

Dessoki et al. reported that beta-thalassemia major patients were significantly more likely to have cognitive deficits than age-matched controls and there was a significant relationship between cognitive deficit and level of hemoglobin and serum ferritin^21^. In contrast in our study, the hemoglobin level was not found to be an associated risk factor of MCI. The reason behinds these discrepancies may be that: (1) the differences in baseline levels of hemoglobin and serum ferritin levels between our study and the study of Dessoki. All of thalassemia patients in our study had lower baseline levels of both hemoglobin and serum ferritin than those reported by Dessoki et al. and (2) the dissimilarity in cognitive testing tools. In our study we used the MoCA test for the detection of cognitive impairment in all alpha- and beta-thalassemia patients, while Dessoki et al. used a computerized Wisconsin’s Card Screening Test to evaluate cognitive performance in only beta-thalassemia major patients. Consistent with the findings of our study, Dessoki et al. reported that the poor cognitive performance of thalassemia patients was not associated with demographic data including sex and the frequency of blood transfusion.

Surprisingly, plasma FGF21 level was significantly increased in thalassemia patients with MCI, compared to patients with non-MCI (Table 2) and showed a significant independent association with a risk of MCI, as shown in Table 3. This observation was consistent with the finding that a high level of FGF21 was associated with a low score of the MoCA test in non-elderly metabolic patients^16^. To add weight to the possible association between FGF21 and cognitive impairment in thalassemia patients, the in vivo results showed that there was an increase in plasma FGF21 and brain FGF21 levels with the reduced expression of brain FGF21 signaling in iron-overloaded thalassemic mice, suggesting a resistance of brain FGF21 might develop in cases of thalassemia with the iron-overload condition. Interestingly, the reduction of brain synaptic protein and Alzheimer’s related proteins have been demonstrated in iron-overloaded thalassemic mice. Collectively, these findings probably indicate that an increase in plasma FGF 21 level show an association with the cognitive decline in thalassemia patients. In addition, we postulated that there may be resistance to FGF21 in thalassemia patients. Any association between FGF21 level and cognitive impairment in thalassemic patients’ needs to be investigated further in a larger population. Furthermore, the role of FGF21 resistance in the thalassemic patient should be explored in future research whether it is the pathogenesis of cognitive impairment or the adaptive process to prevent the effects of the high level of plasma FGF21 in thalassemic patients with cognitive impairment.

Although the level of FGF21 and maximum serum ferritin within 5 years were significantly higher in thalassemia patients with MCI than normal cognitive function, we found that there was no significant correlation between the level of FGF21 and either serum ferritin or maximum serum ferritin within 5 years (Supplementary Fig. 3). Additionally, we did not observe the association between plasma FGF21 level and both ferritin parameters in either NTDT or TDT group (Supplementary Fig. 4). Taken together, these findings suggest that the association of FGF21 level and MCI condition in thalassemia patients was independently related to the severity of iron-overload status (high serum ferritin and maximum level of serum ferritin in the past 5 years).

In summary, the prevalence of MCI was high in thalassemia patients. Plasma FGF21 level was separately related to cognitive dysfunction in thalassemia patients. With the occurrence of brain FGF21 resistance, the reduction of the expression of synaptic proteins and development of Alzheimer’s like pathology in iron-overloaded thalassemia mice model. Increased plasma FGF21 levels in thalassemic patients is associated with cognitive impairment. Therefore, the conditions or medications that reduce plasma FGF21 levels, may help to improve cognitive function in thalassemia patients.

### Study limitation

There were some limitations to this study. First, the study population was relatively small. Second, although our previous study demonstrated that FGF21 level was independently associated with cognitive impairment in non-elderly patients with metabolic syndrome^16^, the association of FGF21 and cognitive function in non-thalassemic subjects in age-matched group with thalassemic patients was not determined in the present study. Therefore, the further study should investigate the association between FGF 21 and cognitive function in non-thalassemic subjects. Third, only the MoCA test was used for the assessment of cognitive function in this study and this test does not evaluate the domain-specific function of cognitive function^22^. However, Positron Emission Tomography/Computed Tomography scans in brain could be further done for detection of brain iron levels and amyloid plaques in thalassemia patients. Finally, brain iron detection using magnetic resonance imaging (MRI) should be used to demonstrate an increase in brain iron levels in thalassemic patients. Unfortunately, we have the limitation in the method of MRI for detection of brain iron. Therefore, the future study to detect brain iron level should be examined in thalassemic patients.

## Methods

### Subject and methods for clinical study

This clinical study was a cross-sectional single-center study. The study protocols were approved by the institutional Ethics Committee of the Faculty of Medicine, Chiang Mai University, Chiang Mai, Thailand (Approval Number: 438/2561). All patients gave written informed consent for research participation. All methods were performed in accordance with the relevant guidelines and regulations.

All of the thalassemia patients (18–60 years old) who attended at the hematology clinic, Maharaj Hospital, Faculty of Medicine, Chiang Mai University, Thailand, from November 2018 to November 2019 were enrolled onto the study. Both alpha- and beta-thalassemia patients with transfusion-dependent and non-transfusion dependent were enrolled.

Exclusion criteria were patients who had neurological disorders, psychiatric problems, a history of brain surgery, and a history of traumatic brain injury. Cognitive function was assessed in all patients using the MoCA test. Demographic data including age, sex, and educational level were collected in addition to thalassemia profile including type and severity of thalassemia, transfusion status, the maximum level of serum ferritin in the past 5 years, and medication taken. Clinical parameters including complete blood count, serum creatinine, electrolytes, liver function test, and serum ferritin, and plasma FGF21 level were also determined in all patients.

We defined a total MoCA score of less than 26 points as mild cognitive impairment (MCI) (Total score was 30 points)^23^. Transfusion dependent thalassemia (TDT) was categorized as thalassemia patients who required regular red blood cell transfusion with no transfusion-free period of more than eight weeks^3,24^. Thalassemia patients not requiring regular red blood cell transfusion for survival but intermittently less than three red blood cell transfusion per year were categorized as non-transfusion dependent thalassemia (NTDT)^4^. Iron overload was specified as a level of serum ferritin more than 1000 mg/dl in TDT patients and more than 800 mg/dl in NTDT patients^3,4^.

### Animal study

**Figure 4:**
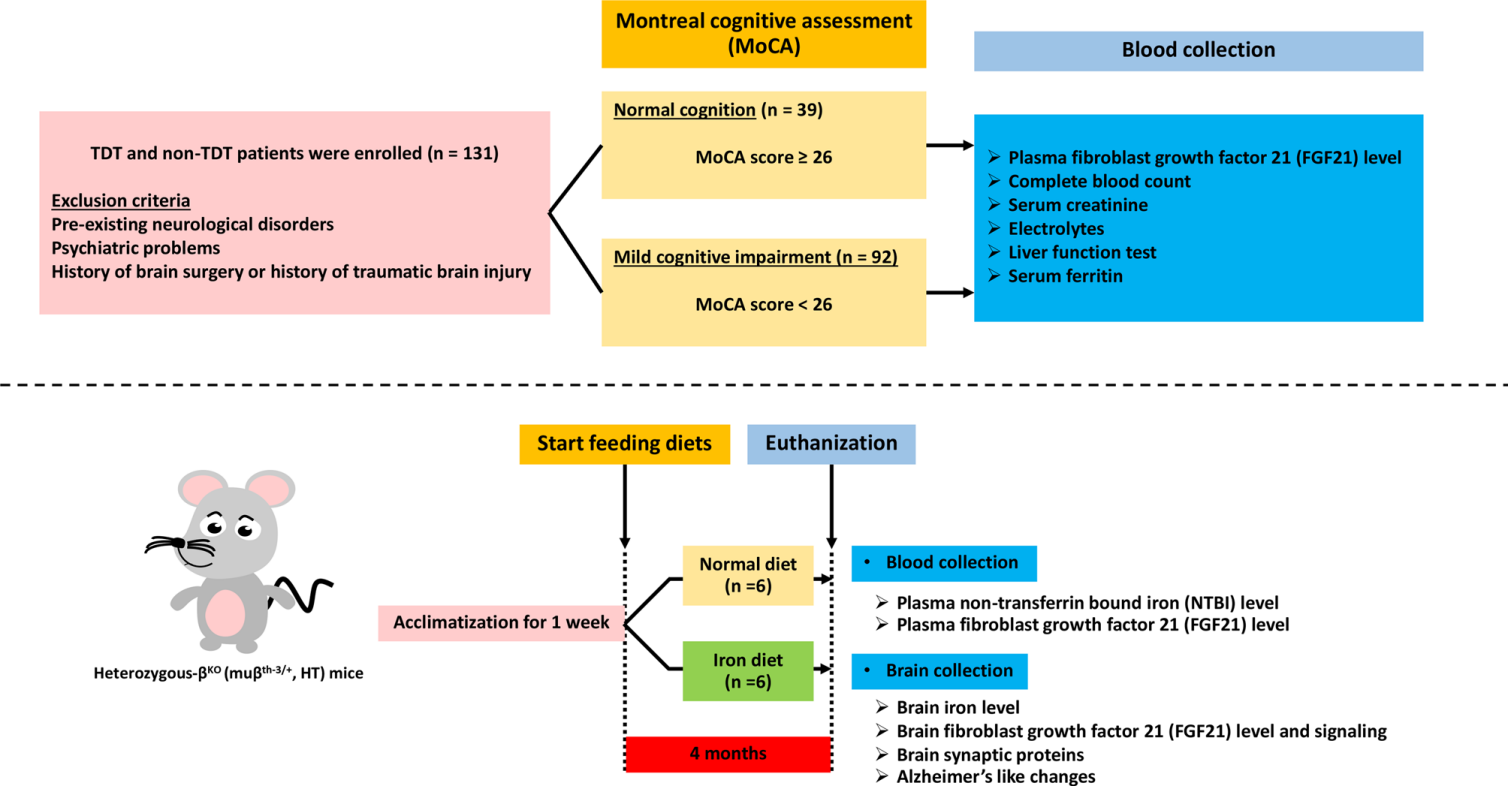
Summarized experimental protocol for clinical and in vivo studies. *TDT* transfusion-dependent thalassemia.

The study protocol for animal study was ethically approved by the Institutional Animal Care and Use Committee from the Faculty of Medicine, Chiang Mai University (Approval number: 2562/MC-0004), and in compliance with the ARRIVE guidelines. Twelve heterozygous β-knockout C57/BL6 mice (muβ^th-3/+^, HT, 3–6 months old) obtained from the Thalassemia Research Center, Institute of Molecular Biosciences, Mahidol University, Bangkok, Thailand were used^25,26^. HT mice were divided into two groups (n = 6/group) including: (1) a control group in which HT mice were fed on a normal diet (ND) and (2) an iron-overload group which was fed on a high-iron diet (HFe, 0.2% ferrocene/kg of diet) for 4 months^8,27,28^. Blood samples were collected to obtain plasma and kept at − 80 °C for investigating plasma non-transferrin bound iron (NTBI)^8^ and plasma FGF21^16^. Moreover, brain tissue was collected to enable the investigation of brain iron level^8^ and brain FGF21 level. Expression of synaptic transmission proteins, FGF21 signaling, and Alzheimer’s like pathology were also investigated. All methods were performed in accordance with the relevant guidelines and regulations. A summary of the experimental protocol from clinical and animal subjects is illustrated in Fig. 4.

### Immunoblotting

Brain protein was extracted as described previously^8,29^. Sample proteins were loaded onto 10% SDS-acrylamide gels and subsequently transferred to a nitrocellulose membrane in a Wet-Tank immunoblotting system (Bio-Rad Laboratories, Hercules, California, USA). After blocking, membranes were kept at 4 °C and exposed overnight to specific antibodies including: (1) β-actin; (2) synaptic markers, synaptophysin and post-synaptic density 95 (PSD-95); (3) FGF21 signaling, fibroblast growth factor receptor 1 (FGFR1) and its phosphorylated form (p-FGFR1_Y654_); and β-klotho; (4) Alzheimer’s like pathology amyloid precursor protein (APP); β-amyloid; tau; and its phosphorylated form (p-Tau_Thr181_). ECL western blotting detection reagent was used to visualize protein bands and densitometry analysis was performed using ImageJ program (NIH, Bethesda, MD).

### Determination of plasma and brain FGF21 level

Plasma level of FGF21 in thalassemia patients was determined using a human FGF21 enzyme-linked immunosorbent assay (ELISA) kit (R&D systems Inc., Minneapolis, MN, USA), whereas FGF21 level in the plasma and brain of thalassemic mice were investigated using a mouse FGF21 ELISA kit (Cusabio Technology LLC, Houston, TX, USA).

### Statistical analysis

For the clinical results, continuous variables were expressed as mean ± standard deviation (SD). Comparisons between groups were made using a Student t-test. The non-normally distributed data were represented as median and interquartile range (IQR) and a Mann–Whitney U test was used to analyze these data. Categorical variables were expressed as frequencies and percentage and comparison between groups was made using Chi-square or Fisher’s exact test. The multivariate analysis of the factors associated with mild cognitive impairment was conduct by binary logistic regression. In the animal study, data were expressed as mean ± standard error of the mean (SEM). Comparisons between the groups were made using a Student t-test. A p-value < 0.05 was considered to be a measure of statistical significance. All statistical calculations were assessed using the Statistical Package for the Social Sciences (SPSS) software version 22 (SPSS Inc. Chicago, IL, USA).

## Supplementary Information

Supplementary Information.
