# Author Correction: An association between fibroblast growth factor 21 and cognitive impairment in iron-overload thalassemia

**DOI:** 10.1038/s41598-023-28995-1

**Published:** 2023-02-01

**Authors:** Wasan Theerajangkhaphichai, Jirapas Sripetchwandee, Sirawit Sriwichaiin, Saovaros Svasti, Nipon Chattipakorn, Adisak Tantiworawit, Siriporn C. Chattipakorn

**Affiliations:** 1 Division of Hematology, Department of Internal Medicine, Faculty of Medicine, Chiang Mai University, Chiang Mai, 50200 Thailand; 2 Neurophysiology Unit, Cardiac Electrophysiology Research and Training (CERT) Center, Faculty of Medicine, Chiang Mai University, Chiang Mai, 50200 Thailand; 3 Department of Physiology, Faculty of Medicine, Chiang Mai University, Chiang Mai, 50200 Thailand; 4 Thalassemia Research Center, Institute of Molecular Biosciences, Mahidol University, Nakhon Pathom, 73170 Thailand; 5 Department of Oral Biology and Diagnostic Sciences, Faculty of Dentistry, Chiang Mai University, Chiang Mai, 50200 Thailand

Correction to: *Scientific Reports* 10.1038/s41598-021-87597-x, published online 13 April 2021

In the original version of the Article contained an error in Figure 3(E), where new representative Western blot image of the APP panel was incorrect. This change does not affect the conclusions of the Article.

The original Figure 3 and accompanying legend appear below.

**Figure 3:**
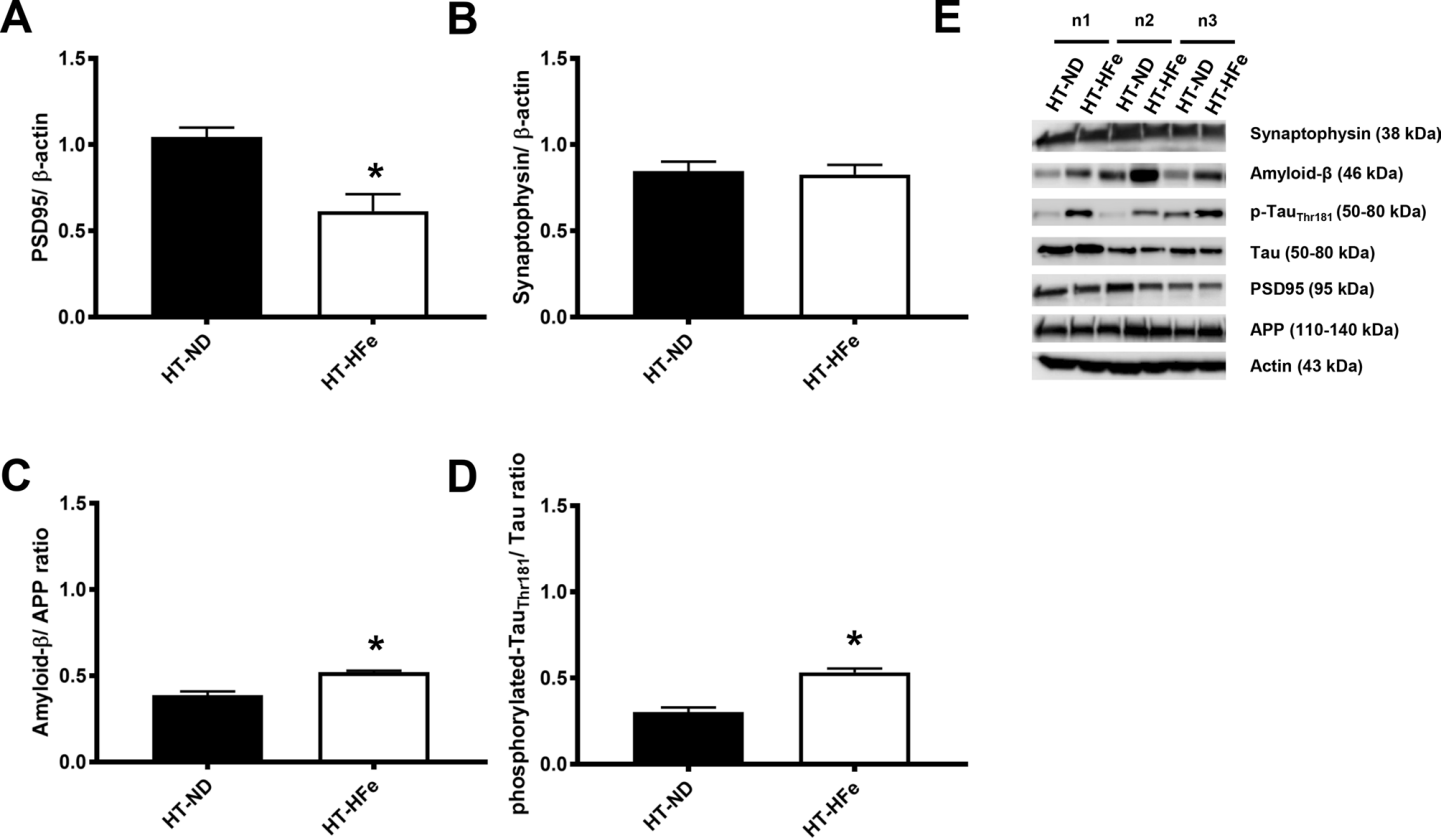
Brain synaptic proteins including PSD95 (**A**) and synaptophysin (**B**) and Alzheimer’s related proteins including the ratios of Amyloid-β/APP (**C**) and phosphorylated-TauThr181/Tau (**D**) of β-thalassemic mice fed with either normal diet or high-iron diet. Three replicates of cropped representative bands for brain FGF21 signaling proteins are shown (**E**). In addition, full-length blots are presented in Supplementary Fig. 2. The samples derive from the same experiment and that gels/blots were processed in parallel. Actin was used as a loading control and the density of detected protein was divided by the density of actin from the sample. *APP* amyloid precursor protein, *HFe* high-iron diet fed mice, *HT* heterozygous-β knockout, *ND* normal diet fed mice, *PSD95* post-synaptic density 95. *p < 0.05 vs. HT-mice fed with normal diet.

The original Article has been corrected.

