# Supplementary material for: An association between fibroblast growth factor 21 and cognitive impairment in iron-overload thalassemia
Source: Sci Rep. 2021 Apr 13;11:8057. doi: 10.1038/s41598-021-87597-x (PMC8044130; doi:10.1038/s41598-021-87597-x)

**Supplementary Information file**

**Title:** An association between Fibroblast Growth Factor 21 and cognitive impairment in iron-overload thalassemia

**Authors:** Wasan Theerajangkhaphichai^1*^, Jirapas Sripetchwandee^2,3*^, Sirawit Sriwichaiin^2,3^,
Saovaros Svasti^4^, Nipon Chattipakorn^2,3^, Adisak Tantiworawit^1#^,
Siriporn C. Chattipakorn^2,3,5#^

**Supplementary figure 1.** Full-length blots of data shown in Figure 3. The gels were initially cut ranged from 35 kDa to 180 kDa. Sizing of proteins on SDS-PAGE was performed by using BLUeye Prestained Protein Ladder (GeneDireX, Inc., Taoyuan, Taiwan). In addition, the samples derive from the same experiment and that gels/blots were processed in parallel. FGF21, fibroblast growth factor 21; FGFR1, fibroblast growth factor receptor 1. Red boxes denoted the regions of the original blots used in the figures 3.

**Supplementary figure 2.** Full-length blots of data shown in Figure 4. The gels were initially cut ranged from 35 kDa to 180 kDa. Sizing of proteins on SDS-PAGE was performed by using BLUeye Prestained Protein Ladder (GeneDireX, Inc., Taoyuan, Taiwan). In addition, the samples derive from the same experiment and that gels/blots were processed in parallel. APP, amyloid precursor protein; PSD95, post-synaptic density 95. Red boxes denoted the regions of the original blots used in the figures 4.

**Supplementary figure 3.** Correlation between plasma FGF21 (lnFGF21) and serum ferritin (A), and maximum serum ferritin within 5 years (B). FGF, fibroblast growth factor.

**Supplementary figure 4.** Correlation between plasma FGF21 (lnFGF21) and serum ferritin (A and C), and maximum serum ferritin within 5 years (B and D) in NTDT patients (upper panels) and TDT patients (lower panels). FGF, fibroblast growth factor; NTDT, non-transfusion dependent thalassemia; TDT, transfusion-dependent thalassemia.


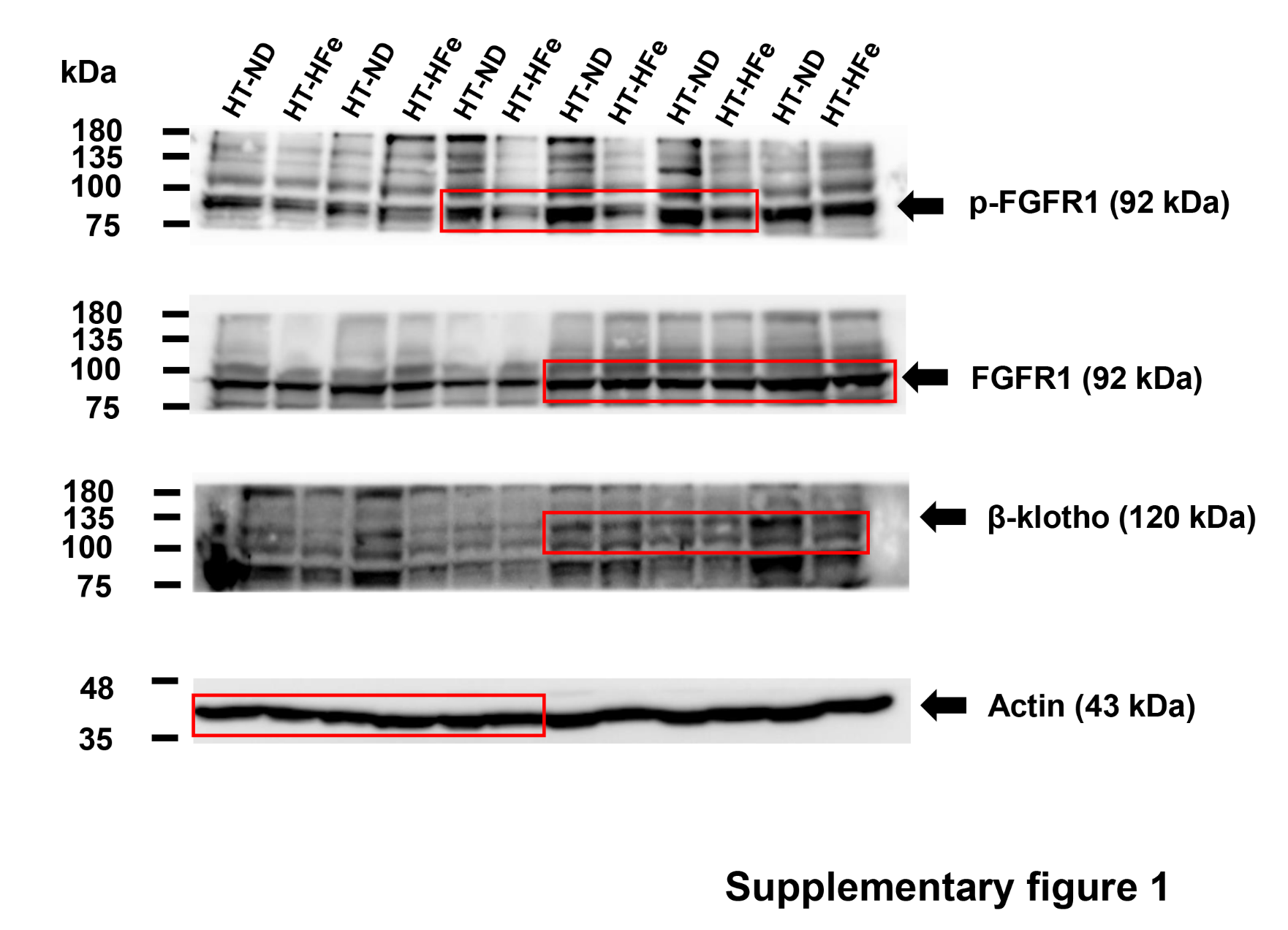


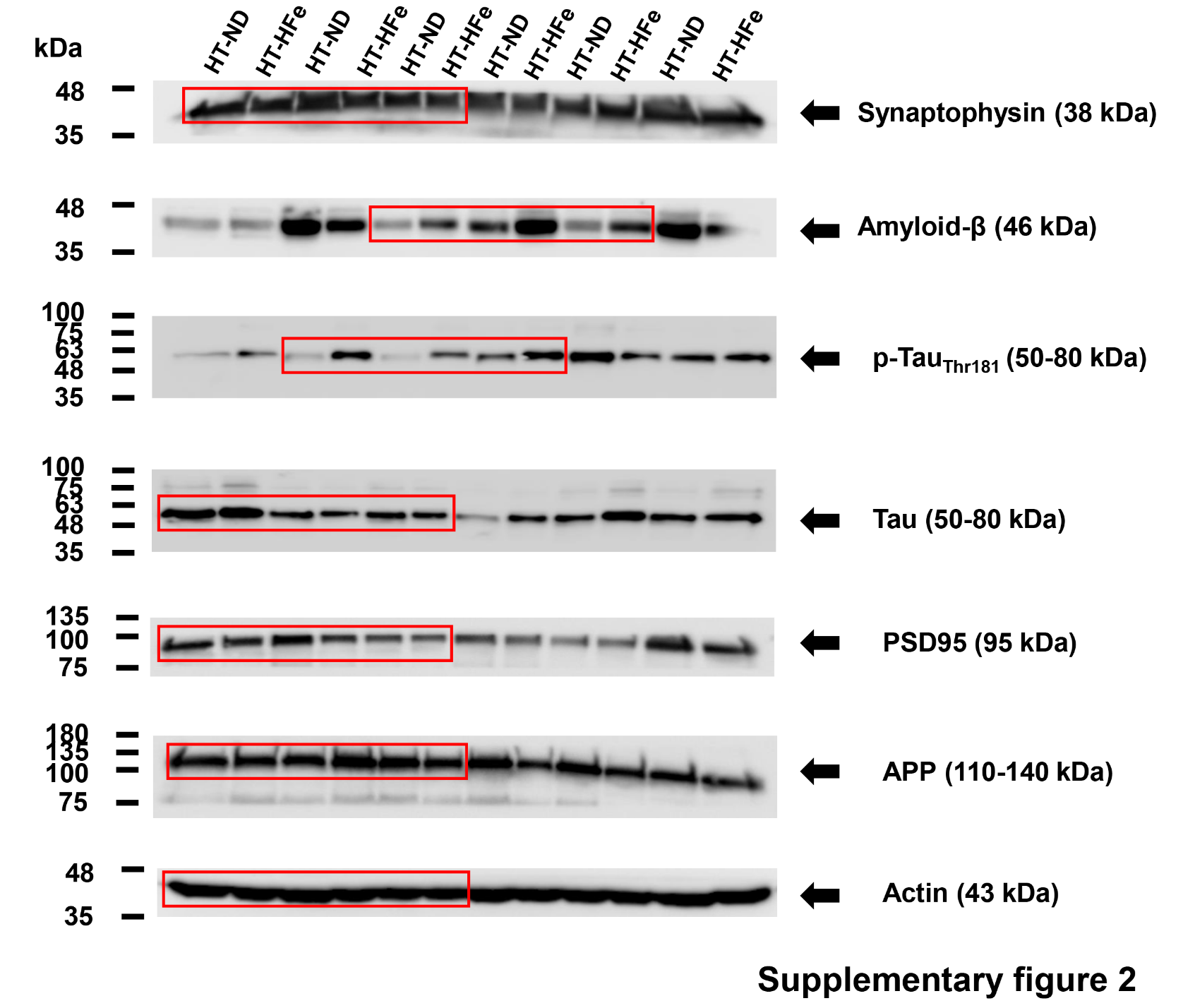


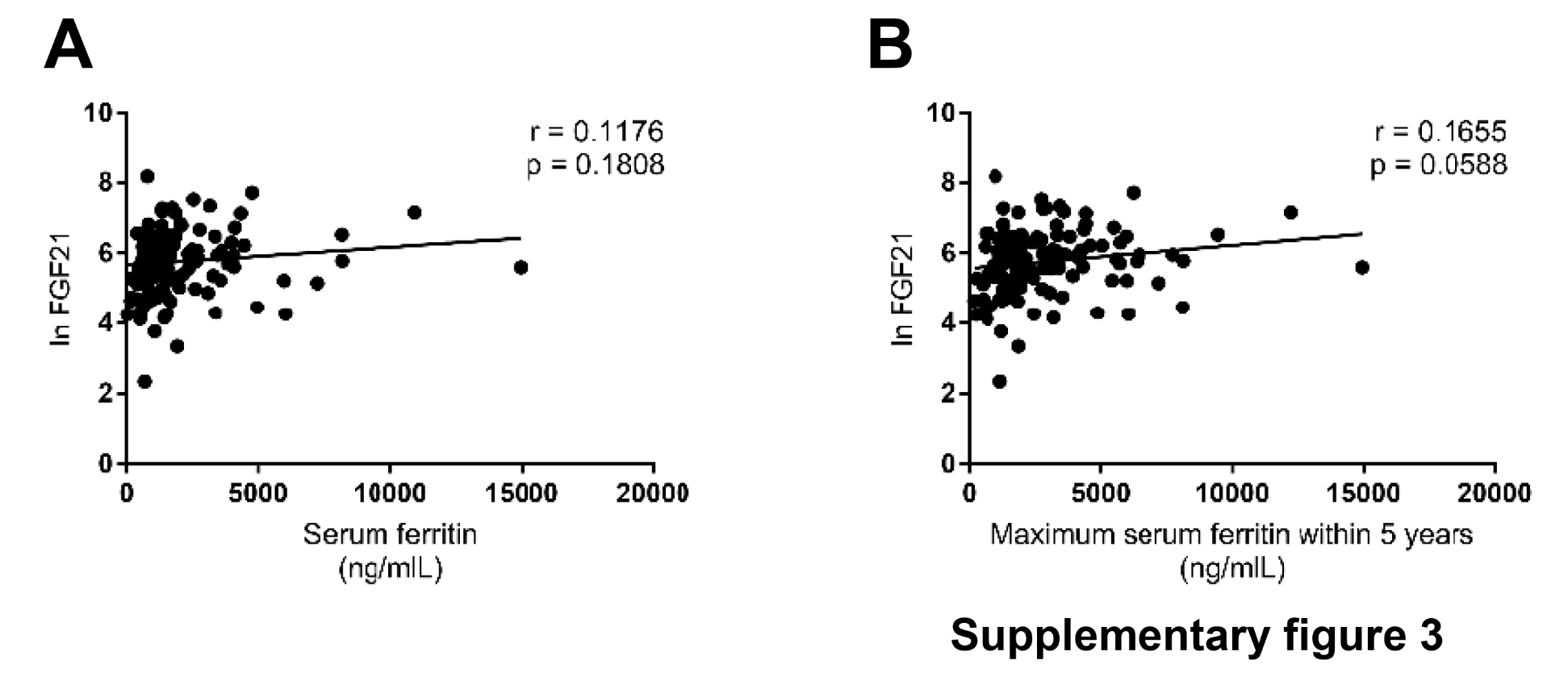


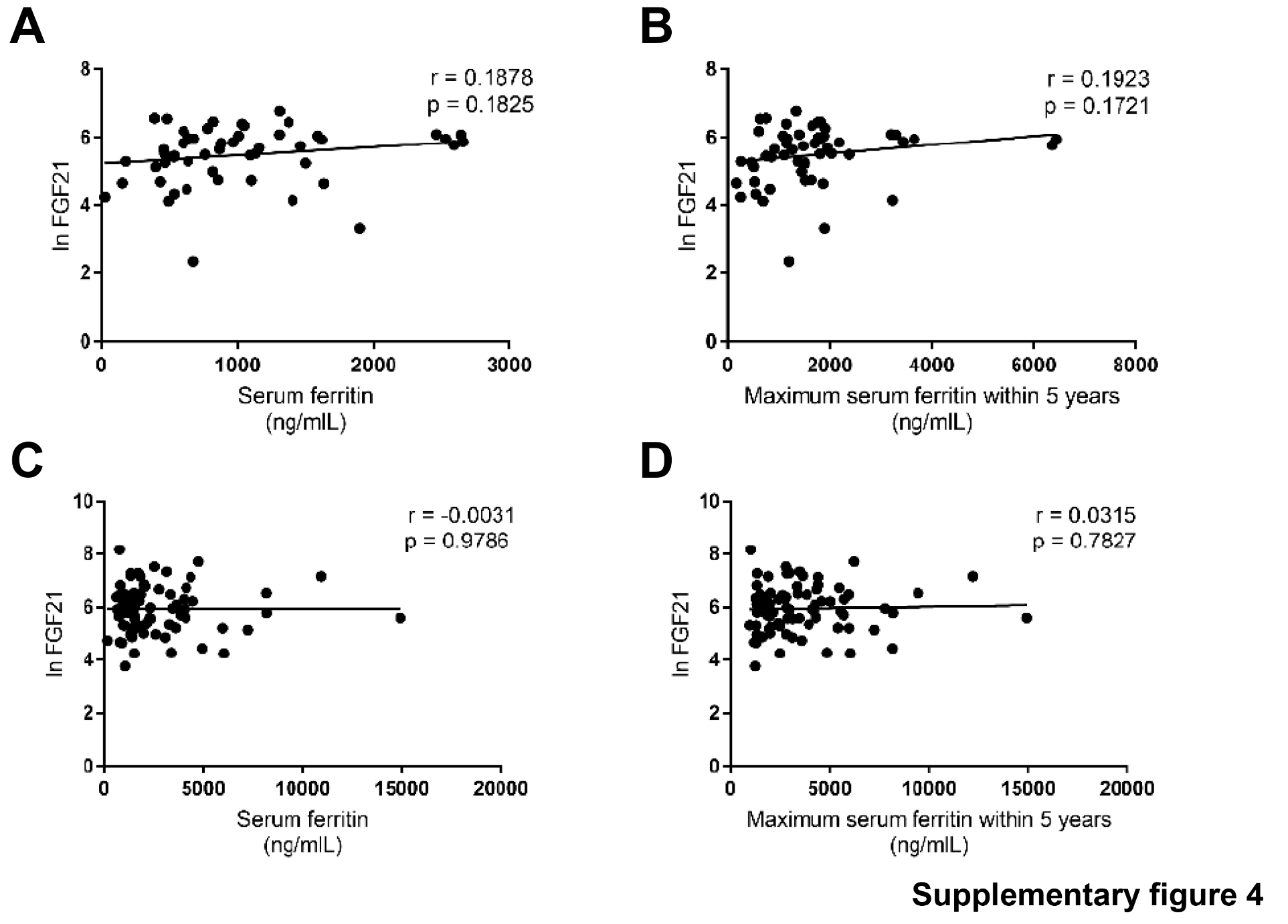

Supplement: Supplementary file 1 — Supplementary Information. [file 41598_2021_87597_MOESM1_ESM.docx]
